# Exploring the Impact of Corporate Social Responsibility Communication through Social Media on Banking Customer E-WOM and Loyalty in Times of Crisis

**DOI:** 10.3390/ijerph18094739

**Published:** 2021-04-29

**Authors:** Dianxi Zhang, Asif Mahmood, Antonio Ariza-Montes, Alejandro Vega-Muñoz, Naveed Ahmad, Heesup Han, Muhammad Safdar Sial

**Affiliations:** 1 Economics and Management School, Wuhan University, Wuhan 430072, China; dxrache@126.com; 2 Department of Business Studies, Namal Institute, Mianwali 42250, Pakistan; asif.mahmood@namal.edu.pk; 3 Social Matters Research Group, Universidad Loyola Andalucía, C/Escritor Castilla Aguayo, 4 14004 Córdoba, Spain; ariza@uloyola.es; 4 Public Policy Observatory, Universidad Autónoma de Chile, 425 Pedro de Valdivia Avenue, Providencia, Santiago 7500912, Chile; alejandro.vega@uautonoma.cl; 5 Faculty of Management Studies, University of Central Punjab, Lahore 54000, Pakistan; naveeddgk2010@gmail.com; 6 College of Hospitality and Tourism Management, Sejong University, 98 Gunja-Dong, Gwanjin-Gu, Seoul 143-747, Korea; 7 Department of Management Sciences, COMSATS University Islamabad (CUI), Islamabad 44000, Pakistan; safdarsial@comsats.edu.pk

**Keywords:** social media, customer loyalty, positive customer emotions, corporate social responsibility, E-word of mouth, banking customers, times of crisis

## Abstract

Previous studies have largely explored corporate social responsibility (CSR) for organization-centric outcomes to achieve organizational performance, organizational commitment, or organizational citizenship behavior. However, the importance of CSR to achieve customer-centric outcomes is underexplored to date. Contemporary researchers have recently turned their attention toward CSR from the viewpoint of customer-centric outcomes. Therefore, the present study attempts to test the influence of CSR communication on social media and customer loyalty in the banking sector of a developing economy in times of crisis. This study also investigates the mediating effect of electronic word of mouth (E-WOM) with this relationship. The data of the present study were collected from different banking customers using a self-administered questionnaire. The empirical findings of this study validated that the CSR communication of a bank on social media increases customer loyalty, and E-WOM partially mediates this relationship. This study will be helpful for the banking sector to understand the importance of CSR communication to increase customer loyalty, which is very important for every bank in times of crisis.

## 1. Introduction

The emergence of social media has facilitated modern business with a digital platform in order to communicate with customers interactively [1]. Social media provides a flexible and interactive forum for businesses in the current age of digitalization [2]. Likewise, social media have revolutionized the field of marketing as the importance of social commerce is being recognized by different contemporary researchers [3,4]. This is perhaps the reason that modern businesses recognize social media as a key player in order to involve different stakeholders, which include involving customers meaningfully with a brand [5]. Social media has brought a substantial change in the field of organizational communication as a new communication medium especially when it takes place from the traditional communication model, which is a one-way model of communication [6]. In the current digital age, the attractiveness of social media is not without logic because its interactive and fast communicability provides an opportunity for businesses to communicate with diverse stakeholders, which is contrary to conventional media [7]. Presently, more than 3.8 billion individuals are already using social media. The attractiveness and acceptability of social media are evident due to the fact that the rate of social media users all over the world is increasing by 7% per year [8]. 

Contemporary businesses use social media for different communication purposes, which include their corporate social responsibility communication (CSR) [9]. There is a stream of researchers who recognize the importance of communicating CSR through social media (S–CSR) as a building block in order to maintain meaningful relationships with stakeholders [10,11,12]. This study defines CSR according to the definition from Carroll [13] who states “CSR is the economic, legal, ethical and philanthropic responsibility of a business.” The extant literature has long established that through well-planned CSR activities, an organization can achieve multiple benefits that include organizational financial performance [14], brand reputation [15], employees’ behavior [16], and organizational commitment [17]. However, how the CSR activities of an organization can generate customer-centric outcomes is an issue that has not received due attention from contemporary CSR researchers thus far. It is quite recent that researchers have turned their attention to investigate the phenomenon of CSR from the perspective of the customers [18,19,20], but these studies are inconclusive, which highlights that there is a need to conduct more research in this field. With this background, the present study attempts to examine the impact of S–CSR on customer loyalty and proposes electronic word of mouth (E-WOM) as a mediator in this relationship.

The study discovered that the banking sector in Pakistan is a suitable segment to test the proposed relationship. The relevance of the banking sector is important for two reasons. First, the banking sector is labeled as a homogenized sector, in which most of the players offer the same type of standard product/services. However, it is very difficult for a bank to earn loyalty from the customers due to this homogenization issue, because loyalty can be earned through different efforts that include a well-differentiated market offering [21], which is almost nonexistent in a homogenized segment. Second, Pakistan’s banking sector is very competitive, which demands some extraordinary and unconventional efforts from a bank to hold its market share. This study argues that the S–CSR of a bank may serve as an extraordinary strategy that can grab the attention of the customers to stay with that bank.

The establishment of a strong brand is critical in services due to service heterogeneity and intangibility [22], and hence, it involves a higher level of purchase risk, compared to physical products [23]. Likewise, service brands possess various touchpoints and customer interactions than brands dealing in the physical product sector [24]. Thus, it is difficult for service brands to portray their image as a socially responsible brand because it is a key strategic touchpoint to develop positive relationships with customers [25]. Hence, the importance to earn an image of a socially responsible brand is increasing for the service sector than ever before [18,26]. Therefore, banks, as one of the players in the service industry, employ various additional strategies to earn a favorable customer attitude [27,28,29,30]. However, it is evident in extant literature that the banking sector received little attention from contemporary scholars to explain how CSR activities of a bank can create positive customer outcomes. Although some attempts have been made by extant researchers [31,32,33,34,35], these studies are sparse, especially from the perspective of developing economies. The above discussion highlights that there is a daunting need to conduct more researches in this area. Furthermore, it is also established in the literature that the relationship between CSR and customer loyalty is complex, and explaining this relationship through a direct influence of CSR on loyalty is not sufficient. In fact, in their recent study, Khan and Fatma [36] mentioned that customers’ CSR perception of an organization is not enough to explain loyalty. Thus, it is better to explain this relationship through moderators and mediators. 

Likewise, there are different important contributions of the present study to the existing literature. First of all, this study adds to the existing CSR literature from the perspective of the customers, whereas the prior studies have largely addressed the relationship of CSR in other domains as opposed to the customers. For example, Barauskaite and Streimikiene [14] investigated CSR in order to enhance the financial performance of an organization. George, Aboobaker, and Edward [17] tested the effect of CSR in order to boost organizational commitment, and Tuan [37] examined CSR from the perspective of employee citizenship behavior. However, the important role of CSR to shape customer emotions and behavior through social media has largely been ignored by contemporary researchers. The present study contends that S–CSR creates an emotional pull (E-WOM) on the part of the customers, which is ultimately translated into a positive customer outcome, i.e., customer loyalty. Lastly, this study is a pioneer to consider the phenomenon of S–CSR in order to achieve marketing-related outcomes, such as customer loyalty and E-WOM, which are very important for every business sector.

## 2. Theory and Hypotheses

The present study uses the lenses of the social exchange theory [38] and the theory of norm reciprocity [39] as the grounding theories. The theory of social exchange proposes that the behavior of individual results from an exchanged process that intends to maximize benefits and reduce costs. Likewise, the theory of norm reciprocity suggests that when an individual receives some benefit from someone, he or she is urged to return that benefit positively. 

Customers make purchase decisions based on their perception of an organizational commitment toward society, the environment, and their analysis of a product/service [40]. When customers see an organization is participating in CSR activities, their positive evaluation of that organization is likely improved [41]. Unfortunately, the idea of CSR has been around for the last two decades in Pakistan, but it is still in the early stages, and the majority of the customers do not have a good level of understanding about the importance of CSR knowledge. The rise of social media is a game-changer from this perspective because it provides an interactive platform for the organizations, which they not only communicate their CSR strategies through with different stakeholders, such as customers, but it also highlights the importance of their CSR initiatives for the community and the environment [42]. Furthermore, CSR initiatives of an organization on social media are also important because of the interactive feature of social media. The customers and the company interactively communicate with each other through social media [43]. This interactive atmosphere of social media plays a key role to build customer confidence for a specific brand, which in turn is translated into increased customers’ loyalty [44]. Different researchers hold the same argument in the context of the banking sector that CSR activities of a bank may induce customer loyalty. For instance, Pérez and Rodríguez del Bosque [45] explored the relationship between customer-centric CSR activities and customer loyalty in the commercial banking sector and found a positive relationship. In different instances, other scholars have also produced the same findings [29,46,47,48].

The customers are more likely to accept products and services from an organization that is a trusted one [49]. The organizations, which are perceived by the customers as socially responsible, are also perceived as trusted organizations. True CSR activities of an organization nurture an environment of transparency, which is very important in order to win customer trust [50]. When customers observe that an organization is contributing positively toward society and the environment through CSR activities, they feel the benefits of CSR overrun the costs, which is in line with the social exchange theory [23]. Likewise, when an organization communicates its CSR activities through social media with the customers, they feel positive and want to reciprocate with the organization positively. Hence, from the perspective of the theory of norm reciprocity [39], S–CSR is expected to earn positive customer outcomes, such as enhanced customer loyalty. As a result, the following hypothesis is postulated.

**Hypothesis** **1** **(H1).** *CSR activities of a bank through social media positively relate to banking customer loyalty*.

Word of mouth (WOM) embraces the informal communication of a customer that is directed at other customers about the characteristics and the experiences of a specific product or brand [51]. Sometimes, WOM has also been recognized as a referral, but it is only one facet of it. Referral means the endorsements of customers about a brand to others [52]. In other words, it is an implicit or an explicit form of recommendation. WOM is regarded as an important element in the field of business, in which WOM customers recommend or do not recommend a brand to others [53]. Research has long established that WOM is a strategic enabler to influence customer loyalty [54,55,56]. E-WOM is the process in which the customers exchange information with each other online about a brand [57]. There have been different researchers in the extant literature, who have acknowledged that E-WOM is positively associated with customer loyalty [58,59]. Prior studies have also established that online communities and forums, customer reviews, and recommendations have transformed digital commerce [60,61]. 

Organizations participate in CSR activities on social media in order to generate an emotional pull among the customers and earn the image of being a socially responsible organization [62]. When social media customers observe that an organization is positively contributing to society and the environment, they feel appreciation for that organization. Hence, the customers want to reciprocate the organization positively, and they are likely to use positive E-WOM about that brand on social media with other customers [63], which is in line with the theory of norm reciprocity [39]. 

Incorporating a CSR program increases the organization’s visibility, and it encourages more communication with the customers. Therefore, it is expected that an organization’s engagement in CSR activities is likely to induce positive WOM on the part of the customers [64]. In the context of the banking sector, Guping et al. [65] validated that CSR activities of a bank help induce WOM for that bank from customers. Similarly, the study of Khan et al. [66] confirmed that CSR activities of a bank are a direct determinant of WOM in the Pakistani banking sector. Different other scholars have also confirmed the positive relationship between CSR activities of a bank and positive WOM [63,67,68].

Customers expect that a responsible organization will provide them with quality products or services without neglecting their social responsibility [69]. The CSR engagement of an organization builds a positive image of the organization [70]. The positive image of an organization that is created by CSR activities leads customers to develop a better perception of the organization through the halo effect. This well-rounded perception of an organization attracts customers to the organization’s products and services [71]. As a result, customers often speak positively about the organization that is engaged in CSR initiatives, and they also associate themselves positively with the products and services of the organization. 

**Figure 1:**
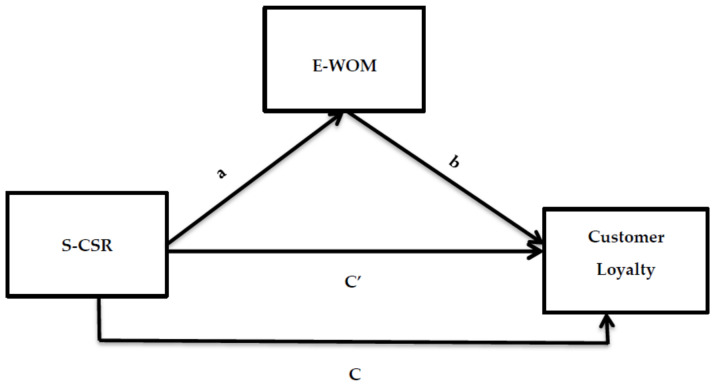
Proposed research model where corporate social responsibility through social media (S–CSR) = the independent variable, customer loyalty = the dependent variable, electronic word of mouth (E-WOM) = the mediating variable, C′ = indirect direct effect of X on Y with the effect of the mediator, and C = direct effect of X on Y without a mediator. a= direct path from S-CSR to E-WOM and b = direct path from E-WOM to customer loyalty.

The role of positive WOM is well recognized among contemporary researchers in order to obtain new customers [72,73,74]. Furthermore, it has also been recognized by recent researchers that positive WOM influences the customer’s positive brand preference [75], perception [76], buying intentions [77], and loyalty [78]. Therefore, WOM is very important for organizations that effectively use WOM in order to increase their sales or have successful promotions. Online media has facilitated WOM online communication with the advent of the internet. Moreover, organizations participate in CSR for several reasons, which include positive WOM [79]. Customers will be more willing to talk about the organization’s CSR activities with their peers, family, and partners as a result of CSR involvement [80]. Modern organizations in the present era use social media to communicate their CSR motives with customers and other stakeholders. In a nutshell, the S–CSR of an organization is expected to engage the customers with a brand meaningfully. In response to the CSR activities of an organization, the customers are self-motivated to support that organization among their social media peers by using positive WOM. All of these activities will ultimately take the customers toward a higher level of loyalty. As a result, the authors propose the following set of hypotheses along with the proposed research model, which is shown in Figure 1.

**Hypothesis** **2** **(H2).** *CSR activities of a bank through social media positively relate to E-WOM for a bank.*

**Hypothesis** **3** **(H3).** *E-WOM mediates the relationship between S-CSR and customer loyalty in the banking sector.*

## 3. Methodology

### 3.1. Population, Sample, and the Handling of Social Desirability 

The authors selected Pakistan’s banking sector to validate the proposed research model, which is shown in Figure 1. For the data collection procedure, the authors first of all intensively assessed the banks that were actively involved in different CSR activities. Furthermore, the authors also verified either the selected banks use social media, such as Facebook, Twitter, Instagram, or YouTube to communicate with their customers. This initial assessment resulted in the selection of four banks, which included Habib Bank Limited (HBL), United Bank Limited (UBL), National Bank of Pakistan (NBP), and Allied Bank Limited (ABL). Likewise, these four banks contain the major share of the banking customers in Pakistan, and they have a presence in almost every city in the country. The authors collected the data from the cities of Lahore, Islamabad, and Karachi. These are all big cities, and they have multiple branches of the banks that were mentioned above in different locations.

The banking sector of Pakistan uses social media to serve different purposes, including communication with stakeholders during the time of crises. Since banking segment is one of those segments that are highly volatile due to different market situation that causes a crisis, these causes may include an excessive risk-taking attitude of a bank, weakness in finance management, changing external market situation, etc. In these instances, the banking institutions need to communicate effectively with their stakeholders, including customers and creditors, to reassure them that their bank will revive soon and will dispose of any crisis-related situation. In this regard, the role of social media, as an interactive medium of communication cannot be neglected because banks communicate their crisis with stakeholders on social media and also share the possible solution and steps taken by the bank to address a crisis-related situation. 

The authors accessed the respondents while they were leaving a particular branch of a bank, or they were found near the ATM premises. Before the collection of the data, the authors received informed consent from each respondent in order to voluntarily participate in the data collection process. Furthermore, the authors also verified that the person who consented to participate in the survey maintained at least one bank account in one of the sample banks mentioned above. Before disseminating the questionnaire among different respondents, they were informed by the authors that they can quit the survey anytime if they feel uncomfortable.

Next, the authors addressed the issue of social desirability by taking different steps. For example, the authors scattered the survey items randomly throughout the questionnaire in order to break any intended sequence of responses regarding answering the questions. Furthermore, this strategy is also helpful to mitigate the impact of any liking or disliking of a specific variable. Likewise, the authors informed the respondents about the importance of genuine response in order to generate the appropriate results from the survey. Additionally, the authors visited different branches of the selected banks during different times; hence, the respondents from all the fields would be included in the survey. The authors distributed 800 surveys among the respondents, and 431 fully completed questionnaires were eventually received, which are included in the final data list. As a result, the response rate of the present survey is 53.87%. 

### 3.2. Measures

In order to address the issue of reliability and validity, the authors used adapted scales to measure all the variables of the present survey. In this regard, the authors borrowed the items of S–CSR from the studies of van Asperen et al. [81] and Eisingerich et al. [82]. This scale contained five items. Similarly, three items that were used to measure E-WOM were taken from the study of Kang and Hustvedt [83]; this scale was also used by Guping et al. [65], in the banking context. Lastly, the three items scale for customer loyalty was adapted from Dagger et al. [84], which is also used by Iglesias et al. [85] in the healthcare insurance context and by Raza et al. [27] in the banking context. A five-point Likert scale was used to record the responses from the different respondents of the present survey. Table 1 illustrates the demographic detail of the respondents. As per the results of Table 1, the gender segment is mainly dominated by males since the majority of the respondents were male who participated in the survey. This dominance is justified in the context of Pakistan, which is a male-dominant society. Likewise, the ages section shows that majority of the respondents were from the age group of 26–30 and 31–40. These insights are also logical and relevant in the context of Pakistan because the first age bracket (20–25) mostly constitute the individuals who are either student or young entrepreneurs at small levels, and they are not frequent bank account users, which is why their portion of frequency is low. One possible reason why the respondents in the last group were low in numbers lies in the logic that the data of the current survey were collected during the period of the COVID-19 crisis in the country, and the majority of the old citizens were not visiting their banks frequently. The detail of all survey items is given in Table 2.

## 4. Results and Analysis

In the data analysis phase, the authors first assessed for the presence of a common method bias (CMB). For this reason, the data for the present survey were collected by the same individual. Hence, the presence of an issue with the CMB is not out of the question. In order to detect a potential issue with the CMB, the authors performed a single-factor analysis according to the guidelines from Harman [86]. In this regard, the authors loaded all the items of variables onto a single factor using IBM-SPSS software version 23. The results of the single-factor analysis validated the absence of any issues with the factors, which shared a total variance of more than 50%. The largest variance shared by a single factor was 39.58%. Therefore, the authors established that there is no issue with the CMB in the present survey. The variance shared by a single factor was 39.58%. Therefore, the authors established that there is no issue with the CMB in the present survey. 

Next, the authors performed an exploratory factor analysis (EFA) by conducting a principal component analysis with a varimax rotation in SPSS in order to detect any cross-item loadings or weak-item loadings. The results showed that the item loadings for all the items were above the threshold level of 0.5 [87]. This validates the data appropriate for further analysis. Table 2 and Table 3 show different results, which include factor loadings, validity analysis, correlation analysis, reliability analysis, and multicollinearity analysis. In this regard, the results of convergent validity (Table 2) were established on the values of the average variance extracted (AVE) for each variable. Additionally, the standard rule here is if the value of AVE for a variable is greater than 0.5, it is established that the criterion for convergent validity is maintained. According to the results from Table 2, all three variables have AVE values greater than 0.5, and therefore, the authors observed no issues with convergent validity. 

The authors also report the discriminant validity results in Table 3, which were obtained by calculating the square root values of AVE for each variable and comparing it with the values of correlation. For instance, the square root value of AVE for the variable loyalty is 0.721, which is larger than the correlation values they were compared to, which are 0.24 ** and 0.31 **. Therefore, it is established that the variables discriminate against each other; hence, the requirement for discriminant validity is fulfilled. The values of composite reliability (CR) were also significant (CR > 0.7). The model fit indices values attained from the confirmatory factor analysis (CFA) are reported in Table 2, (*χ*^2^/*df* = 4.09, RMSEA = 0.0682, NFI = 0.924, CFI = 0.929, and GFI = 0.925). All the results for the model fit indices were in acceptable ranges, which is confirmation that the data are well fitted to the theoretical model. Lastly, the authors reported the results of data normality as per the suggestions from Brown and Dacin [88], who suggested that the data are normally distributed if the skewness and kurtosis values are between ±3 and ±10, which is the case here. 

### Hypotheses Testing 

The authors performed the hypotheses testing for the present study using the structural equation modeling technique (SEM) in IBM-AMOS 21. In this regard, the authors performed the analysis in two steps. In the first step, the authors assessed the results of the direct effect for Hypothesis 1 and Hypothesis 2. The results for the direct effect structural model, which is illustrated in Table 4, revealed that both H1 (H1; *β* = 0.225 **, LLCI = 0.293, ULCI = 0.537, and *p* < 0.05) and H2 (H2; *β* = 0.257 **, LLCI = 0.310, ULCI = 0.583, and *p* < 0.05) are significant and true. Hence, the first two hypotheses, which are denoted as H1 and H2, of the present study are accepted. In the second step, the authors tested the mediating effect of E-WOM between CSR and customer loyalty. The authors used the bootstrapping option in AMOS by using a larger bootstrap sample of 2000. The bootstrapping results confirmed that there is a partial mediation effect of E-WOM between CSR and customer loyalty. The authors confirmed this partial mediation of E-WOM by observing the beta value, which is reduced from 0.225 ** to 0.173 ** but remained positive, which showed that the LLCI and the ULCI are nonzero. All these results confirmed the mediating effect of E-WOM between CSR and customer loyalty. Hence, all three hypotheses of the present study are accepted.

## 5. Discussion and Implications

This study aimed to test the effect of S–CSR on customer loyalty with the mediating effect of E-WOM in Pakistan’s banking sector in times of crisis. In this regard, the empirical findings of the present study confirmed that S–CSR positively influences customer loyalty. The respondents of the present study confirmed that when they are aware of the CSR initiatives of their bank through social media, they have positive feelings, and they want to stay with their bank for a longer time period. The theory of social exchange also justifies this finding since banking customers perceive the CSR practices of a bank in order to create more benefits for society, compared to costs. Hence, they believe that the CSR practices of an organization are helpful to uplift society and the environment. These results revealed that CSR and customer loyalty are positively related. This study is not the first to propose this relationship since various other scholars have also acknowledged that CSR activities of a bank are positively related to banking customer loyalty [29,46,47,48]. This finding can also be explained in the light of social exchange theory because the CSR activities of a bank are well appreciated by the customers when they observe such information on different social media platforms. In exchange, they are urged to hold a higher level of loyalty to a socially responsible bank. The recent studies from different contemporary CSR researchers also provide support for this relationship [9,18,29,89]. 

Moreover, the result of the mediating effect of E-WOM between S–CSR and customer loyalty confirmed that there is a mediating role of E-WOM between this relationship. The theory of norm reciprocity is helpful to explain this result because it shows that when banking customers are informed through different social media platforms about CSR activities of their bank, an emotional pull on the part of the customers is created. In response to this type of emotional pull, the customers are self-motivated to reciprocate their bank positively. Hence, they spread positive WOM about their bank in their social media circles. Eventually, the emotional pull, which is E-WOM, that is created by the CSR initiatives of a bank promotes the customers to a higher level of loyalty. The previous studies showed that the responsible moves of an organization are helpful to create positive emotions on the part of the customers, which are illustrated in the studies by Castro-González et al. [19], and Tajvidi et al. [90]. In addition, it is also evident from the extant researchers that the CSR activities of an organization develop an emotional pull among the customers [91]. From the perspective of social media, the study of D’Acunto et al. [92] is a recent study that acknowledges the importance of S–CSR to create positive emotions among the customers. Different other scholars have also confirmed the existence of a positive relationship between CSR activities of a bank and positive WOM [63,67,68].

There are some important theoretical and practical implications for the present study. First, the study at hand enriches the existing CSR literature from the perspective of the customers, which is very important for every organization. However, the majority of the previous studies have not explored the CSR phenomenon well from the context of the customer-related outcomes. Second, the present study adds to the existing literature by arguing that the S–CSR activities of an organization are helpful to create positive emotions among the customers. In this regard, the previous studies have not adequately addressed the important relationship of CSR to create an emotional pull on the part of the customers. Lastly, the present study is an important contribution to the existing literature from the marketing point of view, because the study argues that the CSR strategies on social media are important to induce the customers’ loyalty. The extant literature surprisingly has not paid due consideration in this domain because the majority of the previous studies addressed CSR in other contexts, such as organizational performance [14], organizational commitment [17], and citizenship behavior [37]. 

This study has some important implications for the practitioners of the banking sector. For instance, this study can help policymakers to improve their understanding of CSR from a marketing perspective. In this regard, the policymakers are encouraged to base their marketing strategies close to the CSR phenomenon, because the well-planned CSR initiatives of a bank can be a source of a stable competitive advantage. Likewise, the policymakers need to realize that using CSR activities to shape the behavior of the customers in a positive manner is not without logic. The banking sector needs to realize that they are dealing with an industry that is mostly homogenized by nature and earning customer loyalty in a segment that is homogenized is very difficult. For this reason, CSR strategies are very important since they may be helpful to grab the loyalty of the customers, which is of the utmost importance for every bank. Another practical implication of this study is that it brings to the surface that policymakers from the banking sector are encouraged to use social media intelligently because it provides an interactive and flexible forum to communicate with their customers. A particular bank is likely to receive a positive evaluation from the customers through the effective utilization of S–CSR, and the customers are self-motivated to promote that bank among their social media peers.

### Limitations and Direction for Future Research

This study also has some limitations. The first limitation of the current study is that understanding human behavior is a difficult task due to its multidimensionality. Hence, explaining the complex customer behavior, which is similar to loyalty, only from the perspective of S–CSR, is not without concern. Therefore, future researchers are encouraged to include more variables in the proposed research model. In this regard, the authors suggest including customer green behavior, customer commitment for sustainability, and organizational image as independent or mediating variables for a better explanation of customer loyalty in future studies. Similarly, another limitation of the present study lies in the nature of the data, because the data are cross-sectional in nature, which limits the ability of the proposed relation(s) for causality. A remedy to address this limitation for future researchers is to use longitudinal data, which are able to explain the causal relationship in a better way, compared to cross-sectional data. Likewise, the present study only considered the positive effect of E-WOM on social media, but it neglected the negative effect of E-WOM on customer loyalty, which is very important to examine. Hence, future researchers need to examine the negative impact of W-WOM on customer loyalty. Furthermore, the present study deals with social media in a positive context, but the reality is that the emergence of social media also brings some challenges, such as information privacy being an issue in social media. Additionally, other challenges include information credibility and ethical issues. Future researchers need to deal with these types of issues in order to find solutions. Last but not the least, considering e-reputation risk for a bank is also an important variable that is not included in the present study, and hence, future researchers are suggested to consider this limitation in their future studies. 

## Figures and Tables

**Table 1:** Demographic information of the sample.

Demographic	Frequency	%
Gender		
Male	288	66.8
Female	143	33.2
**Age**		
20–25	73	16.9
26–30	153	35.5
31–40	138	32.1
Above 40	67	15.5
**Education**		
Intermediate	77	17.9
Graduate	112	26.0
Master	183	42.4
Higher	59	13.7
**Total**	**431**	**100**

**Table 2:** Factor loading, convergent validity, and reliability results.

Variable	Statement	Loading	AVE	CR
S–CSR	I consider this bank a socially responsible bank.	0.71		
	This bank is more beneficial to society’s welfare than other banks.	0.74		
	This bank contributes something to society.	0.79		
	I share this bank’s (CSR) posts on my own Facebook (or other social media) page.	0.83		
	I engage in conversations (CSR) on the Facebook (or other social media) page of this bank.	0.82	0.61	0.88
E-WOM	I am likely to spread positive word of mouth about this bank (on social media).	0.70		
	I would recommend this bank’s products/services to my friends (on social media).	0.77		
	If my friends were looking to purchase banking services, I would tell them to try this bank (on social media).	0.72	0.53	0.77
Loyalty	I consider this bank my first choice when I purchase the services they supply.	0.73		
	I am willing to maintain my relationship with this bank	0.72		
	I am loyal to this bank.	0.84	0.59	0.81

Notes: Loadings = factor loadings, CR = composite reliability, AVE = average-variance-extracted, S–CSR = corporate social responsibility through social medial, E-WOM = electronic word of mouth.

**Table 3:** Correlations and discriminant validity.

Variable		Mean	SD	S–CSR	E-WOM	Loyalty	Skewness	Kurtosis
**S–CSR**		3.88	0.58	**(0.781)**	0.27 **	0.24 **	−0.56	0.47
**E-WOM**		4.10	0.61		**(0.728)**	0.31 **	−0.63	0.51
**Loyalty**		4.28	0.55			**(0.721)**	−0.68	0.44
	(*χ*^2^/*df* = 4.09, RMSEA = 0.068, NFI = 0.924, CFI = 0.929, GFI = 0.925)							

Notes: Bold diagonal = square root of average variance extracted (AVE), ** = values are significant.

**Table 4:** Results of the hypotheses testing.

Path	Beta Value	S.E	LLCI	ULCI	Decision
The Results of Hypothesis 1 and 2					
S–CSR → Loyalty	0.225 **	0.0442	0.293	0.537	supported
S–CSR → E-WOM	0.257 **	0.0371	0.310	0.583	supported
(*χ*^2^/*df* = 3.394, RMSEA = 0.0578, NFI = 0.946, CFI = 0.949, and GFI = 0.947) ***					
**The Results of Hypothesis 3**					
S–CSR → E-WOM → Loyalty	0.173 **	0.021	0.044	0.063	supported
(*χ*^2^/*df* = 2.98, RMSEA = 0.0486, NFI = 0.952, CFI = 0.958, and GFI = 0.956) ***					

S–CSR, CSR on social media; E-WOM, electronic word of mouth; S.E = standard error, LLCI = lower limit confidence interval, ULCI = upper limit confidence interval, and *** and ** = significant values.

## Data Availability

The datasets used in this research are available upon request from the corresponding author. The data are not publicly available due to restrictions, i.e., privacy or ethics.
